# Molecular Characterization and Prevalence of *Anaplasma ovis* and *Anaplasma phagocytophilum* in Goat Population in Siirt Province From Türkiye

**DOI:** 10.1002/vms3.71093

**Published:** 2026-07-21

**Authors:** Muhammed Ahmed Selcuk, Tamer Karakuş, Rustu Erdem Ozen, Ceylan Ceylan, Onur Ceylan

**Affiliations:** ^1^ Faculty of Veterinary Medicine, Department of Parasitology Siirt University Siirt Türkiye; ^2^ Faculty of Veterinary Medicine, Department of Parasitology Selcuk University Konya Türkiye

**Keywords:** Anaplasmataceae, caprine anaplasmosis, phylogeny, Rickettsiales, southeastern Türkiye

## Abstract

Anaplasmosis is a major tick‐borne infectious disease affecting small ruminants, leading to significant economic losses due to decreased productivity and, in some cases, mortality. This study aimed to determine the molecular prevalence of *Anaplasma ovis* and *Anaplasma phagocytophilum* infections in goats raised in Siirt province, Türkiye, and to genetically characterize the detected isolates based on the major surface protein 4 (*MSP4*) gene region. A total of 250 randomly selected apparently healthy goats were sampled between July and September 2025. The overall molecular prevalence of *A. ovis* was found to be 52% (130/250), while *A. phagocytophilum* was not detected in any of the screened animals. Significant associations were observed between *A. ovis* positivity and age, sex, breed, and sampling districts within Siirt province (*p* < 0.05), with the highest infection rates observed in goats older than 1 year (63.6%), female goats (58.2%), Damascus goats (66.7%), and goats sampled from the Kurtalan district (87.1%). Sequencing analysis of selected samples yielded high‐quality *MSP4* gene sequences showing 100% nucleotide identity with reference *A. ovis* isolates from Iran, Italy, Pakistan, Sudan, Tunisia, and Türkiye. Phylogenetic analysis supported species‐level identification by grouping the study isolates with reference *A. ovis* sequences; however, broader evolutionary inferences should be interpreted cautiously due to the short fragment length and limited number of sequenced isolates. The findings of this study provide updated molecular epidemiological data on caprine anaplasmosis in southeastern Türkiye and highlight the utility of the *MSP4* gene as a reliable molecular marker for species identification and phylogenetic analysis. These results contribute to a better understanding of the epidemiology of *A. ovis* and may support the development of effective control strategies in small ruminant production systems.

## Graphical abstract

This study assessed the molecular prevalence and genetic characterization of *Anaplasma ovis* and *Anaplasma phagocytophilum* in goats from Siirt using MSP4‐based PCR and sequencing; a high prevalence of *A. ovis* (52.0%) was detected with significant risk factor associations, while *A. phagocytophilum* was not identified; phylogenetic analysis showed that the isolates were identical to global strains, confirming the reliability of the *MSP4* gene as a molecular marker.

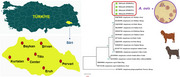

## 1 Introduction

Anaplasmosis is a significant vector‐borne infectious disease that causes substantial economic losses, decreased productivity, and occasionally mortality in animal husbandry. It is caused by obligate intracellular pathogens belonging to the family Anaplasmataceae, within the class α‐proteobacteria and the order Rickettsiales. These pathogens primarily infect erythrocytes but may also parasitize other blood cells in mammals and are mainly transmitted by ixodid ticks of the genera *Ixodes*, *Dermacentor*, *Rhipicephalus*, and *Amblyomma* (Kocan et al. 2000; Dumler et al. 2001; Rymaszewska and Grenda 2008).

Anaplasmosis is an important infectious disease in small ruminant production, primarily caused by *Anaplasma ovis* and *Anaplasma phagocytophilum*, and is associated with significant economic losses. Although it often follows a subclinical course, it can lead to evident clinical manifestations and substantial production losses under favourable conditions (Ceylan, Xuan, et al. 2021). These pathogens, particularly in the presence of stress, immunosuppression, or concurrent infections, may cause anaemia, anorexia, weight loss, growth retardation, and poor body condition, ultimately resulting in marked reductions in meat, milk, and wool production. Even subclinical infections have been reported to adversely affect herd performance, while clinical cases may be associated with abortion, fertility disorders, and, in rare instances, mortality (Dumler et al. 2001; Alessandra and Santo 2012). Maintained in nature through infected animals and ixodid tick vectors, these pathogens acquire an enzootic character through the reservoir–vector relationship; moreover, the broad host range and zoonotic potential of *A. phagocytophilum* increase the importance of the disease not only in veterinary medicine but also from a public health perspective. When considered together with treatment costs, production losses, and decreased productivity, this results in substantial economic consequences in small ruminant farming (Dumler et al. 2005; Garcia‐Perez et al. 2003; Alessandra and Santo 2012).

*Anaplasma ovis* infections in sheep and goats were initially identified through microscopic examination of peripheral blood smears; in subsequent years, the presence of *A. phagocytophilum* was confirmed in various animal species, particularly sheep, cattle, goats, and dogs, using PCR‐based molecular methods, allowing a more detailed understanding of the epidemiology and genetic diversity of these pathogens (Dumler et al. 2001; de la Fuente et al. 2007; Benedicto et al. 2020). However, the morphological similarity between *Anaplasma* and *Ehrlichia* species, together with the occurrence of cross‐reactions in serological tests, complicates their definitive differentiation using conventional diagnostic approaches (Bradway et al. 2001; Stoltsz 2004). Therefore, especially in cases of low parasitaemia and in asymptomatic carrier animals, PCR‐based molecular diagnostic methods with high sensitivity and specificity have become increasingly important for the reliable detection of these pathogens (Torina et al. 2012; Chi et al. 2013; Ceylan and Ekici 2023).

Currently, the major surface protein 4 (*MSP4*) gene region is widely recognized as a reliable molecular marker for the identification and genetic characterization of *Anaplasma* species. Owing to its conserved structural features across different *Anaplasma* species, the *MSP4* gene serves as a suitable target for amplification and comparative analyses, while the nucleotide polymorphisms it harbours enable discrimination at both species and strain levels. For these reasons, *MSP4* is extensively used not only for species identification but also in phylogenetic analyses, population genetics studies, and the assessment of geographical variation (de la Fuente et al. 2005; Smrdel et al. 2015; Ceylan, Byamukama, et al. 2021; Köseoğlu et al. 2024).

The aim of this study was to determine the presence of *A. ovis* and *A. phagocytophilum* infections in goats raised in the Siirt province using molecular methods, to genetically characterize the obtained isolates based on the *MSP4* gene region, and to provide up‐to‐date data on the prevalence of these infections in the region, where limited molecular epidemiological information is currently available despite favourable ecological conditions for tick‐borne pathogen transmission.

## 2 Material and Methods

### 2.1 Study Area, Animals, and Blood Samples

**FIGURE 1:**
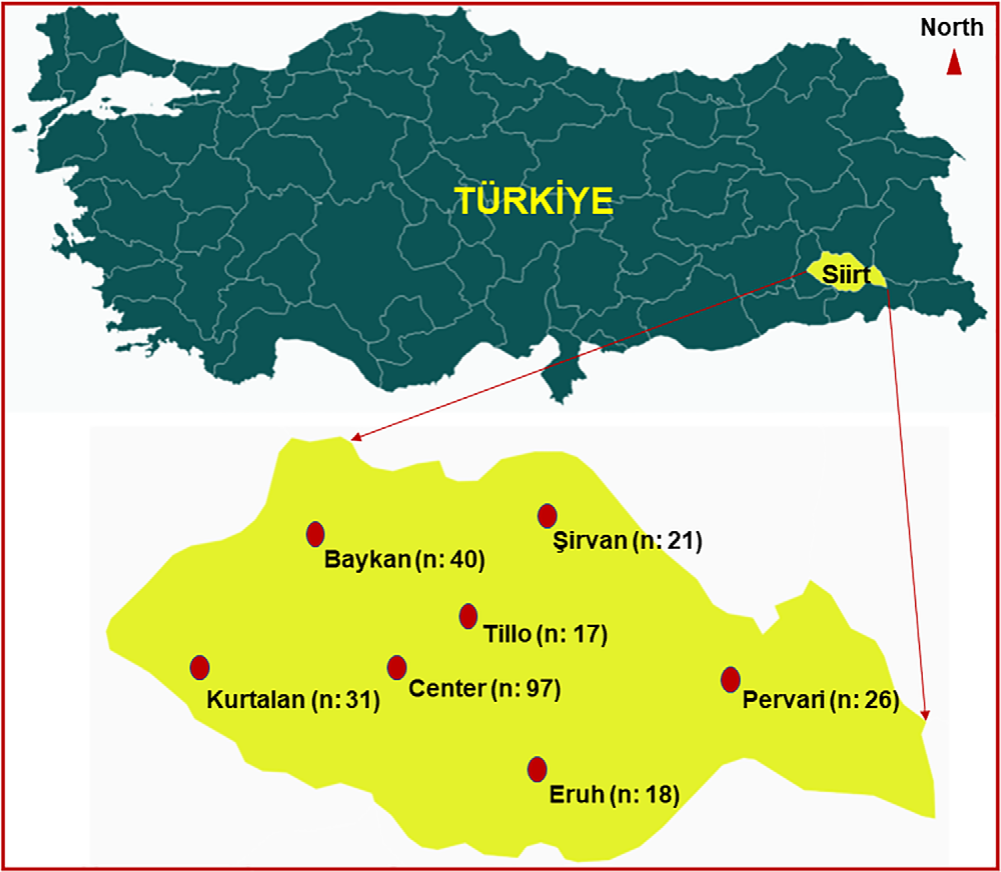
Map showing the Siirt province, including the city centre and its districts, where goat samples were collected, along with the number of animals sampled in each location.

In this study, blood samples were collected between July and September 2025 from goats raised under traditional farming conditions in Siirt province and its districts, located in the Southeastern Anatolia region of Türkiye, during the period of intense tick activity. A district‐based stratified sampling approach was adopted, in which multiple herds from each district were included, and goats within herds were randomly selected. Herd owners/farms were selected based on both random selection and voluntary participation. To improve representativeness across districts and herds, approximately 10% of the animals within each accessible herd were sampled proportionally. Accordingly, the final sample size (*n* = 250) was determined considering herd population, field accessibility, and proportional representation of the study area. A total of 250 randomly selected apparently healthy goats without evident clinical signs at the time of sampling were included in the study, consisting of 63 animals aged ≤1 year and 187 animals aged >1 year, 213 females and 37 males, and 205 Hair goats and 45 Damascus goats. The animals were sampled from different districts as follows: Baykan (*n* = 40), Eruh (*n* = 18), Central district (*n* = 97), Kurtalan (*n* = 31), Pervari (*n* = 26), Şirvan (*n* = 21), and Tillo (*n* = 17) (Figure 1). Blood samples were collected via jugular venipuncture using sterile syringes and transferred into EDTA‐containing tubes at a volume of 5 mL. Information on location, sex, age, and breed of the animals was recorded. The blood samples were transported to the laboratory under cold chain conditions and stored at −20°C until molecular analyses were performed.

### 2.2 Genomic DNA Extraction and PCR Amplification of the *MSP4* Gene

Genomic DNA (gDNA) was extracted from blood samples using a commercial Diagen DNA Extraction Kit (Diagen, Ankara, Türkiye). The obtained DNA samples were stored at −20°C until use in molecular analyses. The *MSP4* gene region was targeted for the detection of *A. ovis* and *A. phagocytophilum* in the extracted gDNA samples. For the identification of *A. ovis*, species‐specific primer sets F (5′‐TGAAGGGAGCGGGGTCATGGG‐3′) and R (5′‐GAGTAATTGCAGCCAGGGACTCT‐3′) were used to amplify a 347‐bp fragment (Torina et al. 2012). For the detection of *A. phagocytophilum*, primer sets F (5′‐ATGAATTACAGAGAATTGCTTGTAGG‐3′) and R (5′‐TTAATTGAAAGCAAATCTTGCTCCTATG‐3′) were used to amplify an 849‐bp fragment (de la Fuente et al. 2005). PCR reactions were performed in a total volume of 25 µL, consisting of 10 µL A.B.T. 2X PCR Master Mix (ABT Laboratory Industry, Türkiye), 1 µL each of forward and reverse primers (20 pmol/µL), 2.5 µL template DNA, and 10.5 µL nuclease‐free distilled water (ddH_2_O). The PCR thermal cycling conditions for *A. ovis* included an initial denaturation at 94°C for 10 min, followed by 30 cycles of denaturation at 94°C for 30 s, annealing at 62°C for 15 s, and extension at 72°C for 30 s, with a final extension at 72°C for 5 min. For *A. phagocytophilum*, the PCR programme consisted of an initial denaturation at 94°C for 10 min, followed by 40 cycles of denaturation at 94°C for 30 s, annealing at 60°C for 45 s, and extension at 72°C for 1 min, with a final extension at 72°C for 7 min. PCR products were subjected to electrophoresis on 1.4% agarose gel at 100 V for 30 min, and the bands were visualized under UV light using a gel documentation system (Erbiotek, Türkiye). A 100‐bp DNA ladder (ELK Biotechnology, China) was used to determine the molecular size of the bands. Samples showing clear band profiles were sent to a commercial company (BM Labosis, Türkiye) for unidirectional DNA sequencing analysis.

### 2.3 Sequencing and Phylogenetic Analysis

The quality control of the obtained sequence data was performed using FinchTV v1.4.0 (Geospiza Inc., Seattle, WA, USA). Sequences with acceptable read quality were compared with reference sequences in the National Center for Biotechnology Information (NCBI) database using the Basic Local Alignment Search Tool (BLAST) for species confirmation. The sequences confirmed at the species level were then imported into the MEGA X software for multiple sequence alignment. Following alignment, sequence quality was visually assessed in MEGA X, and low‐quality or unreadable nucleotide regions at the beginning and end of the sequences were trimmed to avoid potential bias in downstream analyses. After trimming and sequence editing, a final alignment length of 288 bp was obtained for phylogenetic analysis. Gaps and missing data were handled using the partial deletion option in MEGA X. The edited sequences were subsequently analyzed together with homologous reference sequences retrieved from the NCBI database. Based on the model selection analysis conducted in MEGA X, the Kimura 2‐parameter model with invariant sites (K2+I) was determined as the most suitable nucleotide substitution model, and the phylogenetic tree was constructed using the maximum likelihood (ML) method with 1000 bootstrap replicates (Kumar et al. 2018).

### 2.4 Statistical Analysis

Statistical analyses were performed using the SPSS software (version 25.0). The associations between *A. ovis* positivity and categorical variables (age, sex, breed, and location) were evaluated using the Pearson chi‐square (*χ* ^2^) test. When more than 20% of the expected cell counts were less than five, Fisher's exact test or Monte Carlo simulation was applied as appropriate. Factors associated with *A. ovis* infection were identified using univariable and multivariable binary logistic regression analyses. Odds ratios were reported together with 95% confidence intervals. A *p* value of <0.05 was considered statistically significant.

## 3 Results

### 3.1 Molecular Prevalence and Statistical Analysis Findings

The overall molecular prevalence of *A. ovis* was 52% (130/250). A statistically significant association was found between *A. ovis* positivity and age, sex, breed, and location (*p* < 0.001, *p* < 0.001, *p* = 0.044, and *p* < 0.001, respectively). The prevalence was significantly higher in goats older than 1 year (63.6%) compared to those ≤1 year (17.5%). Female goats showed a higher prevalence (58.2%) than males (16.2%). In terms of breed, Damascus goats had a higher prevalence (66.7%) compared to Hair goats (48.8%). Additionally, significant differences were observed among locations, with the highest prevalence in Kurtalan (87.1%) and the lowest in Şirvan (4.8%). Detailed data on molecular prevalence and statistical analyses are provided in Table 1.

**TABLE 1:** Distribution and prevalence (%) of *Anaplasma ovis* infection according to sex, age, breed, and the districts where the samples were collected, as well as the levels of statistical significance (p values).

Variable	Group	Positive/total	Prevalence (%)	*p* value
Age	≤1 year	11/63	17.5	<0.001
	>1 year	119/187	63.6	
Sex	Female	124/213	58.2	<0.001
	Male	6/37	16.2	
Breed	Hair goat	100/205	48.8	0.044
	Damascus	30/45	66.7	
Location	Centre	46/97	47.4	<0.001
	Kurtalan	27/31	87.1	
	Pervari	12/26	46.2	
	Şirvan	1/21	4.8	
	Eruh	8/18	44.4	
	Baykan	27/40	67.5	
	Tillo	9/17	52.9	
Total		130/250	52.0	

*Note*: *p* values indicate the level of statistical significance for comparisons between groups. Statistical significance was accepted at *p* < 0.05.

According to the univariable logistic regression analysis evaluating factors associated with *A. ovis* infection in goats from Siirt province, goats older than 1 year had 8.3 times higher odds of *A. ovis* infection compared with goats aged ≤1 year (odds ratio [OR] = 8.273, *p* < 0.001). Female goats had 7.2 times higher odds of infection than males (OR = 7.199, *p* < 0.001), whereas Damascus goats had 2.1 times higher odds compared with Hair goats (OR = 2.100, *p* = 0.032). When the odds of *A. ovis* infection were evaluated according to districts using Siirt Central district as the reference category, the odds were 7.5 times higher in Kurtalan (OR = 7.484, *p* < 0.001) and 2.3 times higher in Baykan (OR = 2.303, *p* = 0.034), whereas they were 94.5% lower in Şirvan district (OR = 0.055, *p* = 0.006). According to the multivariable logistic regression analysis, goats older than 1 year had 13.1 times higher odds of *A. ovis* infection (adjusted odds ratio [aOR] = 13.062, *p* < 0.001), while female goats and Damascus goats had 6.2‐fold (aOR = 6.161, *p* < 0.001) and 22.1‐fold (aOR = 22.106, *p* < 0.001) higher odds, respectively. When district‐level differences were assessed relative to Siirt Central district, the odds of *A. ovis* infection were 12.5 times higher in Kurtalan (aOR = 12.529, *p* < 0.001), 10.9 times higher in Pervari (aOR = 10.929, *p* < 0.001), 6.8 times higher in Baykan (aOR = 6.859, *p* < 0.001), and 5.4 times higher in Tillo (aOR = 5.442, *p* = 0.013) (Table 2).

**TABLE 2:** Univariable and multivariable logistic regression analyses of factors associated with *A. ovis* infection in goats.

	Univariable		Multivariable	
	OR (95% CI)	*p* value	Adjusted OR (95% CI)	*p* value
Age				
≤1 year	Ref.			
>1 year	8.273 (4.045–16.919)	**<0.001**	13.062 (4.584–37.221)	**<0.001**
Sex				
Male	Ref.			
Female	7.199 (2.881–17.984)	**<0.001**	6.161 (2.090–18.160)	**<0.001**
Breed				
Hair goat	Ref.			
Damascus	2.100 (1.066–4.135)	**0.032**	22.106 (6.475–75.471)	**<0.001**
Location				
Centre	Ref.			
Kurtalan	7.484 (2.434–23.009)	**<0.001**	12.529 (3.727–42.117)	**<0.001**
Pervari	0.950 (0.399–2.264)	0.908	10.929 (2.847–41.954)	**<0.001**
Şirvan	0.055 (0.007–0.429)	**0.006**	0.242 (0.028–2.098)	0.198
Eruh	0.887 (0.323–2.439)	0.816	2.112 (0.661–6.751)	0.207
Baykan	2.303 (1.064–4.985)	**0.034**	6.859 (2.592–18.151)	**<0.001**
Tillo	1.247 (0.444–3.502)	0.675	5.442 (1.439–20.583)	**0.013**

*Note*: Bold values represent statistically significant odds ratios (*p* < 0.05).

Abbreviations: CI, confidence interval; OR, odds ratio; Ref, reference category.

### 3.2 Analysis of *MSP4* Gene Sequences

As a result of molecular screening using species‐specific primers, no amplification products indicative of *A. phagocytophilum* were detected in the samples included in this study. In contrast, the 347‐bp fragment of the *MSP4* gene region specific to *A. ovis* was successfully amplified, and distinct bands of the expected size were observed (Figure S1).

Following PCR amplification, four samples that yielded clear, specific, and high‐quality bands were subjected to unidirectional sequencing for further molecular characterization. The obtained sequences were aligned with reference *A. ovis* sequences available in the NCBI GenBank database. During alignment, trimming was performed by removing unreadable or low‐quality nucleotide regions at the beginning and end of the sequences. After these quality control and editing steps, reliable sequences of 288 bp in length were obtained for each sample. BLAST analysis of the edited sequences in the NCBI GenBank database revealed that all samples showed 100% nucleotide identity with reference *A. ovis* isolates. This result confirmed the species‐level identification of the amplified *MSP4* gene region and supported the reliability of the molecular data obtained. Furthermore, the sequences generated in this study were submitted to the NCBI GenBank database for public access; the isolates were designated as TRSiAo01–TRSiAo04 and assigned the accession numbers PX982911–PX982914, respectively.

### 3.3 Phylogenetic Analysis Findings

**FIGURE 2:**
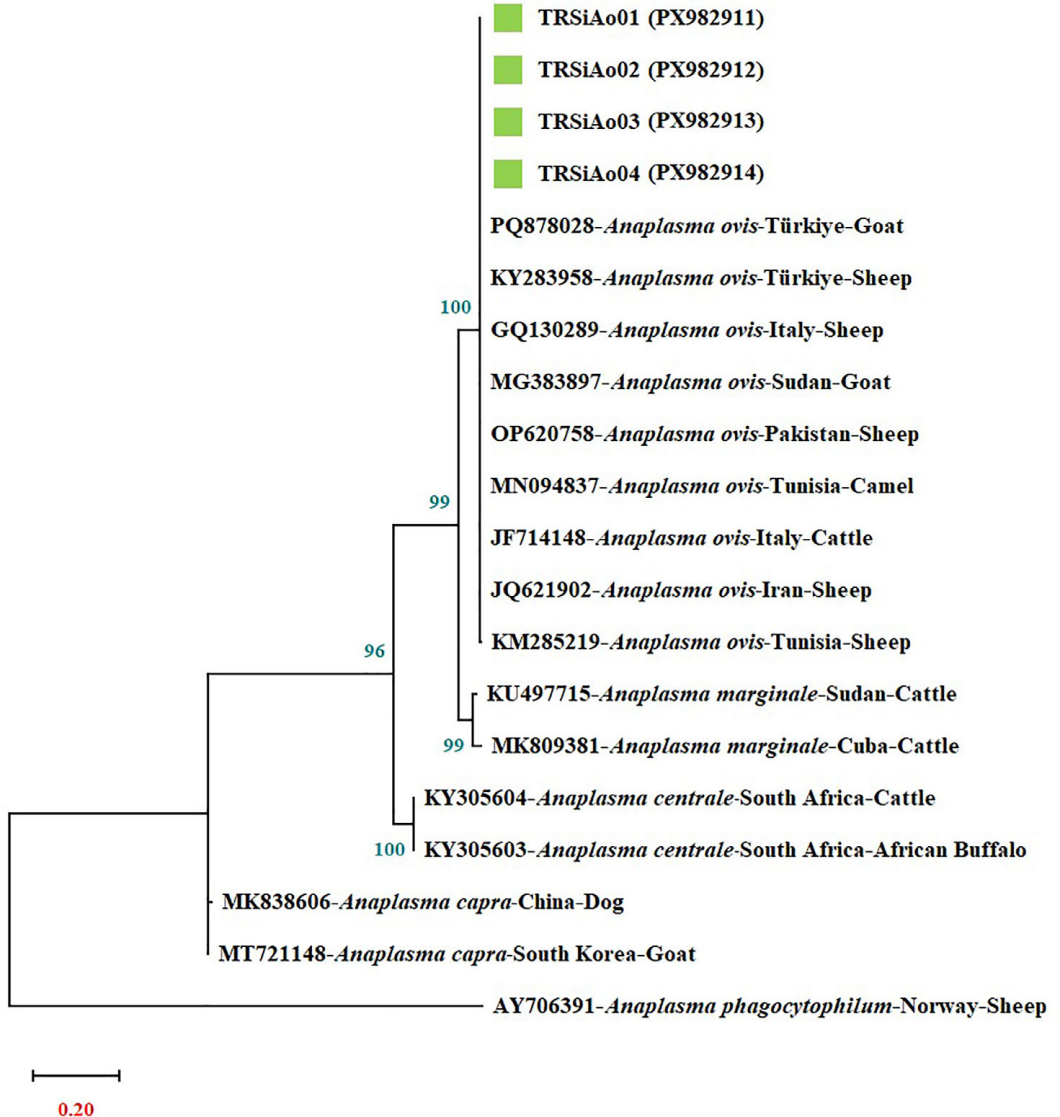
Phylogenetic tree constructed using the 288 bp *Anaplasma ovis MSP4* gene sequences obtained in this study and reference sequences retrieved from GenBank. Phylogenetic analysis was performed using the Kimura 2‐parameter model with invariant sites (K2+I) and the maximum likelihood (ML) method in MEGA X software. The *MSP4* gene sequence of *Anaplasma phagocytophilum* (AY706391) was used as an outgroup to root the phylogenetic tree.

The *MSP4* gene sequences of the molecularly characterized *A. ovis* isolates were used for species identification by comparison with reference sequences (PQ878028, KY283958, GQ130289, MG383897, OP620758, MN094837, JF714148, JQ621902, and KM285219) showing high similarity in BLAST analysis. Phylogenetic analysis based on the *MSP4* gene region revealed that the A. ovis isolates obtained in this study clustered within the same phylogenetic clade together with reference A. ovis isolates reported from different geographical regions, and this clustering was supported by high bootstrap values. For comparative purposes, sequences of other *Anaplasma* species retrieved from the NCBI GenBank database (A. marginale [KU497715, MK809381], A. centrale [KY305604, KY305603], and A. capra [MT721148, MK838606]) were also included in the analysis. These sequences were observed to form distinct and well‐supported clades, clearly separated from the A. ovis cluster. To root the phylogenetic tree, the *MSP4* gene sequence of *A. phagocytophilum* (AY706391) was used as an outgroup, and this sequence was found to be clearly distinct from *Anaplasma* species, ensuring reliable tree rooting (Figure 2).

## 4 Discussion

Sheep and goat farming in Türkiye constitutes one of the fundamental components of the livestock sector and is particularly concentrated in the Eastern and Southeastern Anatolia regions (Taşkın and Kandemir 2022). In these regions, the extensive use of seasonal grazing and transhumance‐based husbandry systems, involving the movement of herds between pastures, provides favourable ecological conditions for the transmission and persistence of various parasitic diseases (Yalçın 1986; Vasileiou et al. 2015; Ceyhan et al. 2017). In Siirt province, where goat farming plays a significant role in regional livestock production, the adverse effects of these infections on animal health and productivity lead to substantial economic losses; consequently, there has been a noticeable increase in the number of scientific studies conducted in this field (Ceylan et al. 2025; Kara et al. 2025; Selcuk et al. 2026). Therefore, determining the presence and prevalence of *Anaplasma* species in the goat population of Siirt province is of great importance for elucidating the epidemiological characteristics of these infections and for developing region‐specific control strategies.

In this study, the prevalence of *A. ovis* in goats from the Siirt province was determined to be 52%. This rate is generally consistent with the prevalence range of 15.1%–71.2% reported in small ruminants in Türkiye using molecular methods (Altay et al. 2014; Öter et al. 2016; Bilgic et al. 2017; Zhou et al. 2017; Aktas and Özübek 2018; Sevinc et al. 2018; Benedicto et al. 2020; Ceylan, Byamukama, et al. 2021; Gökmen et al. 2025; Oguz and Deger 2025). The prevalence obtained in the present study was higher than those reported by Aktas and Özübek (2018) for small ruminants in Adana, Mersin, Gaziantep, and Adıyaman (21.8%, 16.7%, 17.9%, and 15.9%, respectively), as well as the 42.51% prevalence reported by Öter et al. (2016) in goats from the Thrace region. In contrast, it was lower than the prevalence rates of 60% reported by Zhou et al. (2017) in Konya and Karaman, 63.3% reported by Bilgic et al. (2017) in apparently healthy small ruminants, 67.06% reported by Altay et al. (2014) in small ruminants from Eastern Anatolia, and 71.2% reported by Oguz and Deger (2025) in goats from Van province. When compared with international data, the prevalence observed in this study was lower than those reported in goats in Iran (63.73%) and in Spain (91.1%, determined by reverse transcription polymerase chain reaction [RT‐PCR]) but higher than those reported in sheep in Italy (37%), goats in Uganda (25.4%), Saudi Arabia (15.5%), and asymptomatic goats in Pakistan (15%) (Ahmadi‐Hamedani et al. 2009; Torina et al. 2010; Shabana et al. 2018; Kasozi et al. 2021; Lacasta et al. 2021; Taqddus et al. 2023). These findings indicate that *A. ovis* infection exhibits a wide range of prevalence both in Türkiye and worldwide and that the rate detected in the Siirt province is consistent with the existing literature, representing a noteworthy but not exceptional level.

When the distribution of *A. ovis* infection in Siirt province was evaluated, the prevalence was found to vary significantly according to age, sex, breed, and location. The higher infection rate observed in goats older than 1 year (63.6%) compared to those 1 year of age or younger (17.5%) may be associated with prolonged exposure to infected ticks and cumulative contact over time. This observation was further supported by logistic regression analysis, in which goats older than 1 year showed significantly increased odds of *A. ovis* infection in both univariable (OR = 8.273) and multivariable (aOR = 13.062) models, indicating age as an important independent predictor of infection. Similarly, it has been reported that age is an important risk factor for *Anaplasma* spp. infections in small ruminants, with prevalence increasing in older age groups. In terms of sex, the prevalence detected in female goats (58.2%) was significantly higher than that in males (16.2%), which is consistent with previous studies reporting higher infection rates in females and may be associated with physiological or management‐related factors; however, reproductive status, including pregnancy and lactation, was not evaluated in the present study (Cheah et al. 1999; Belkahia et al. 2015; Onyiche et al. 2022). Similarly, logistic regression analysis demonstrated that female goats had markedly higher odds of infection than males in both univariable (OR = 7.199) and multivariable (aOR = 6.161) models, suggesting that sex remained independently associated with *A. ovis* positivity even after adjustment for potential confounding variables.

With respect to breed, the prevalence in Damascus goats (66.7%) was higher than that in Hair goats (48.8%), and this difference was statistically significant; although some studies have reported breed‐related differences in prevalence, this relationship is not consistently observed across all investigations, and therefore the observed variation may be associated with differences in husbandry practices, environmental exposure, and vector contact between breeds, although these factors were not specifically evaluated in the present study and therefore should be interpreted cautiously (Eisawi et al. 2020; Arif et al. 2023). Notably, although the breed effect appeared modest in the univariable model (OR = 2.100), multivariable logistic regression demonstrated substantially higher adjusted odds of infection in Damascus goats (aOR = 22.106), suggesting that potential confounding factors may have influenced the crude association between breed and infection risk. Regarding location, the highest prevalence was recorded in the Kurtalan (87.1%) and Baykan (67.5%) districts, while the lowest prevalence was observed in the Şirvan district (4.8%), and these inter‐district differences may be associated with regional ecological and management‐related factors, including variation in vector exposure; however, tick density, vector distribution, pasture management, and environmental risk factors were not investigated in the present study. District‐level variation was also supported by multivariable logistic regression analysis, in which goats from Kurtalan, Pervari, Baykan, and Tillo showed significantly higher adjusted odds of infection compared with the central district. These findings may suggest a possible influence of local ecological and management‐related factors on transmission dynamics. Previous studies have likewise indicated that variations in *A. ovis* prevalence across different regions may be associated with tick abundance, bioclimatic and ecological conditions, farm management, and husbandry practices (M'ghirbi et al. 2022; Cardillo et al. 2025). Overall, these findings demonstrate that *A. ovis* infection in Siirt province not only exists but also exhibits a heterogeneous epidemiological distribution that varies depending on host‐related factors and regional conditions.

The absence of molecular detection of *A. phagocytophilum* in goats from the Siirt province appears to be consistent with previously reported low‐prevalence data in small ruminants in Türkiye. Indeed, molecular prevalence rates for this pathogen in the country have been reported to range from 0% to 66.7%; however, positivity rates in goats are generally low (Gokce et al. 2008; Altay et al. 2014; Öter et al. 2016; Sevinc et al. 2018; Bilgic et al. 2017; Zhou et al. 2017; Benedicto et al. 2020; Ceylan and Ekici 2023; Gökmen et al. 2025). In the global literature, it has likewise been reported that the prevalence in small ruminants varies considerably depending on the region (Yang et al. 2016; Song et al. 2020; Noaman 2022). Nevertheless, a negative molecular result does not necessarily indicate the complete absence of the pathogen but may instead reflect a very low level of infection or the lack of detectable circulation during the sampling period. Furthermore, factors such as the target gene used, the molecular method applied, and genetic variation among circulating strains may also influence the results. Therefore, the negative finding obtained in this study should be interpreted within the context of the low‐prevalence data reported in Türkiye as well as the regional variability emphasized in the literature.

Morphological similarities and serological cross‐reactions among *Anaplasma* species infecting small ruminants limit the specificity of conventional diagnostic methods; therefore, molecular approaches based on PCR and sequence analysis are considered the primary tools for reliable species‐level identification (Dumler et al. 2001; Stoltsz 2004; de la Fuente et al. 2002; Aktas et al. 2009). In this context, the *MSP4* gene is widely recognized as a reliable molecular marker for the diagnosis and phylogenetic analysis of *Anaplasma* species, as it enables the differentiation of genetic variation among species (de la Fuente et al. 2002; Contreras et al. 2017).

BLAST analysis performed using the GenBank database revealed that the *MSP4* gene sequences obtained from goat samples showed 100% nucleotide identity with reference *A. ovis* sequences, strongly confirming the accurate species‐level identification of the isolates. The fact that the *MSP4* gene possesses discriminative features among species and exhibits a highly conserved structure within *A. ovis* isolates indicates that this gene region represents a reliable target for molecular diagnosis and genetic characterization studies (de la Fuente et al. 2002; Selmi et al. 2020; Oguz and Deger 2025). Similarly, studies conducted in Türkiye and in various geographical regions have reported high sequence similarity between *A. ovis* *MSP4* sequences and reference strains (Dülek et al. 2025; Gökmen et al. 2025; Oguz and Deger 2025). In contrast, no amplification of *A. phagocytophilum* was obtained in the analyzed samples, which may suggest that the pathogen is absent from the studied population, present at a low prevalence, or associated with regional vector ecology.

Phylogenetic analysis revealed that the *A. ovis* isolates obtained in this study clustered within the same clade as reference sequences based on the *MSP4* gene region, with strong grouping supported by high bootstrap values. The clear and consistent separation of the study isolates from other *Anaplasma* species (*Anaplasma marginale*, *Anaplasma centrale*, *Anaplasma capra*, and *Anaplasma phagocytophilum*) in the phylogenetic tree demonstrates that they were accurately identified at the species level and that their phylogenetic positions are reliable. These findings are consistent with previous studies reporting that phylogenetic analyses based on the *MSP4* gene region exhibit high discriminatory power in distinguishing *Anaplasma* species (Yousefi 2018; Cabezas‐Cruz et al. 2019; Selmi et al. 2020; Gökmen et al. 2025).

This study has several limitations that should be acknowledged. First, tick collection and vector identification were not performed; therefore, the contribution of specific tick species to pathogen transmission and the possible role of vector‐related ecological differences between districts could not be evaluated. In addition, several potentially relevant host‐ and farm‐level factors, including reproductive status, acaricide use, pasture management, herd movement, and other management‐related practices, were not assessed. Clinical findings and haematological parameters were also unavailable, preventing the evaluation of the potential clinical impact of infection. Furthermore, because the study employed a cross‐sectional design, the observed associations should not be interpreted as causal relationships. The unequal distribution of animals across sex, breed, and district categories, together with the lack of herd‐level clustering analysis, may also have influenced some risk‐factor estimates. Therefore, the epidemiological associations identified in the present study should be interpreted cautiously, and future longitudinal studies integrating vector, management, clinical, and environmental data are warranted to better clarify the epidemiology and transmission dynamics of caprine anaplasmosis in the region.

## 5 Conclusion

The detection of a high molecular prevalence of *A. ovis* in goats in Siirt province revealed that this pathogen is widespread in the region and poses a significant infection risk. In contrast, the absence of *A. phagocytophilum* suggests that this species is either not present in the investigated population or circulates at very low levels. These findings contribute to a better understanding of the epidemiological structure of anaplasmosis in goats in Siirt province and provide an important basis for the development of region‐specific control strategies. However, further advanced molecular and epidemiological studies involving larger sample sizes, different seasons, and potential vectors are recommended to more comprehensively elucidate the true distribution of the infection in the region.

## Author Contributions

**Tamer Karakuş**: methodology, software, investigation, writing – review and editing, project administration. **Onur Ceylan**: validation, formal analysis, supervision, writing – original draft, writing – review and editing, resources, data curation, visualization. **Rustu Erdem Ozen**: methodology, software, investigation, writing – review and editing, project administration. **Muhammed Ahmed Selcuk**: conceptualization, methodology, software, validation, formal analysis, investigation, resources, data curation, writing – original draft, writing – review and editing, visualization, project administration, funding acquisition. **Ceylan Ceylan**: validation, formal analysis, resources, data curation, writing – original draft, writing – review and editing, supervision.

## Funding

The authors have nothing to report.

## Ethics Statement

This study was carried out with the approval of the Siirt University Experimental Animals Application and Research Center Animal Experiments Local Ethics Committee (decision No: 2025/04/29).

## Conflicts of Interest

The authors declare no conflicts of interest.

## Supporting information




**Supplementary Figure 1**. Agarose gel electrophoresis image of the PCR amplification of the *MSP4* gene region of *Anaplasma ovis*. M: Marker (100 bp), P: Positive control, 1–4: *A. ovis*‐positive goat isolates, N: Negative control.

## Data Availability

The datasets generated and/or analyzed in this study are publicly available in the National Center for Biotechnology Information (NCBI) GenBank database (https://www.ncbi.nlm.nih.gov/) under the accession numbers PX982911–PX982914.
