# Supplementary figures and images for: Molecular Characterization and Prevalence of Anaplasma ovis and Anaplasma phagocytophilum in Goat Population in Siirt Province From Türkiye
Source: Vet Med Sci. 2026 Jul 21;12(4):e71093. doi: 10.1002/vms3.71093 (PMC13387591; doi:10.1002/vms3.71093)

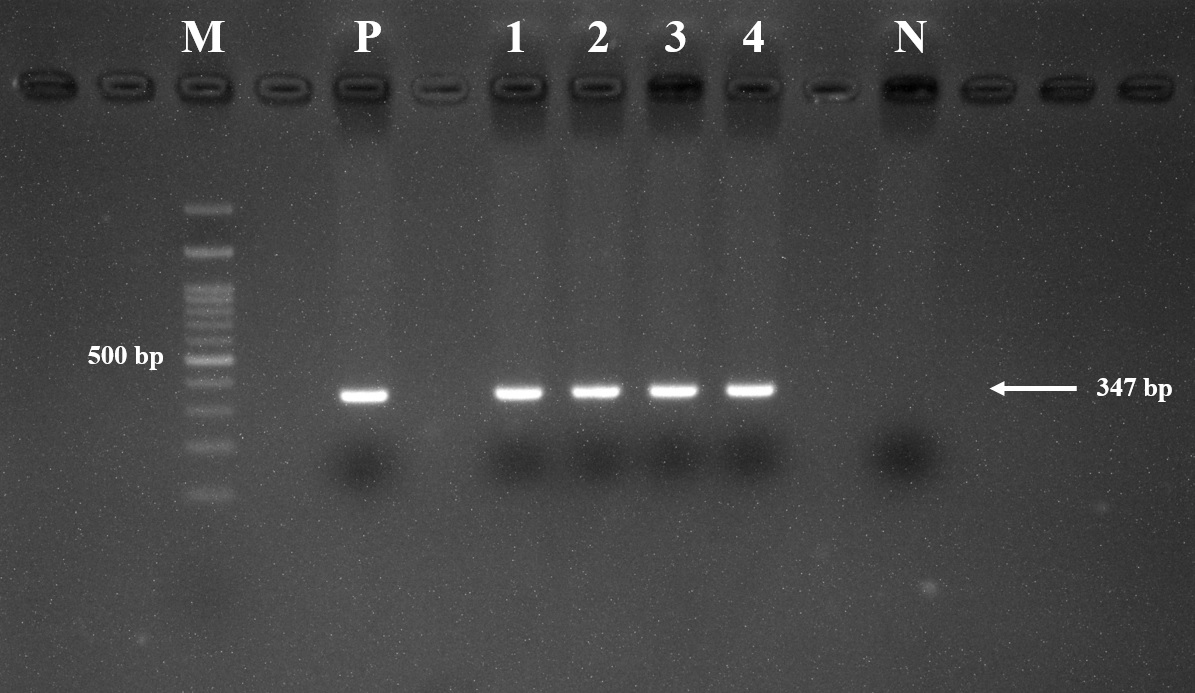

Supplement: Supplementary file 1 — Supplementary Figure 1. Agarose gel electrophoresis image of the PCR amplification of the MSP4 gene region of Anaplasma ovis. M: Marker (100 bp), P: Positive control, 1–4: A. ovis‐positive goat isolates, N: Negative control. [file VMS3-12-e71093-s001.jpg]
